# Acute Failure of a Glenoid Component in Anatomic Shoulder Arthroplasty

**DOI:** 10.1155/2016/6208294

**Published:** 2016-07-31

**Authors:** William E. Daner, III, Norman D. Boardman, III

**Affiliations:** Department of Orthopaedic Surgery, Virginia Commonwealth University Health System, Richmond, VA 23298, USA

## Abstract

Glenoid loosening is the most common cause of failure in primary total shoulder arthroplasty (TSA) and often occurs years after the initial surgery. It is rare for a glenoid component to fail acutely. Several case reports of complete glenoid dissociation appear in the literature. It is important to report these failures to identify technical errors or component design flaws to improve outcomes in TSA. In this case report, we present an unrecognized acute failure of a cemented hybrid glenoid component at the time of surgery.

## 1. Introduction

Total shoulder arthroplasty (TSA) is an increasingly common procedure in the treatment of omarthrosis, with more than 50,000 total shoulder arthroplasties being performed annually in the United States [1]. This number has been on the rise since the early 1990s, particularly since FDA approval of reverse total shoulder arthroplasty (RTSA) for use beginning in 2004 [2]. Anatomic TSA comprises the majority of these cases, the most common indication being primary osteoarthritis [2]. TSA has proven to be an excellent pain relieving procedure that can improve range of motion and restore function. The overall complication rate has been reported to range from 12 to 39.8%, and the majority of these complications are related to glenoid component loosening [3–5]. Patients with a failed TSA may present with a painful, stiff shoulder with decreased range of motion, with or without associated symptoms of instability.

Several case reports of complete glenoid dissociation appear in the literature [6–9]. Among these failures were metal-backed components in which the polyethylene liner dissociated from the glenoid baseplate anywhere from 7 to 50 months postoperatively [7–9]. One report involved failure of a cemented all-polyethylene component 12 months after primary TSA [6]. Analogous failures have occurred through the pegs of all-polyethylene patella components in total knee arthroplasty, none acutely [10–12]. To the best of the authors' knowledge, there are no reported cases of acute failure of a cemented glenoid component at the time of surgery.

## 2. Case Report

**Figure 1:**
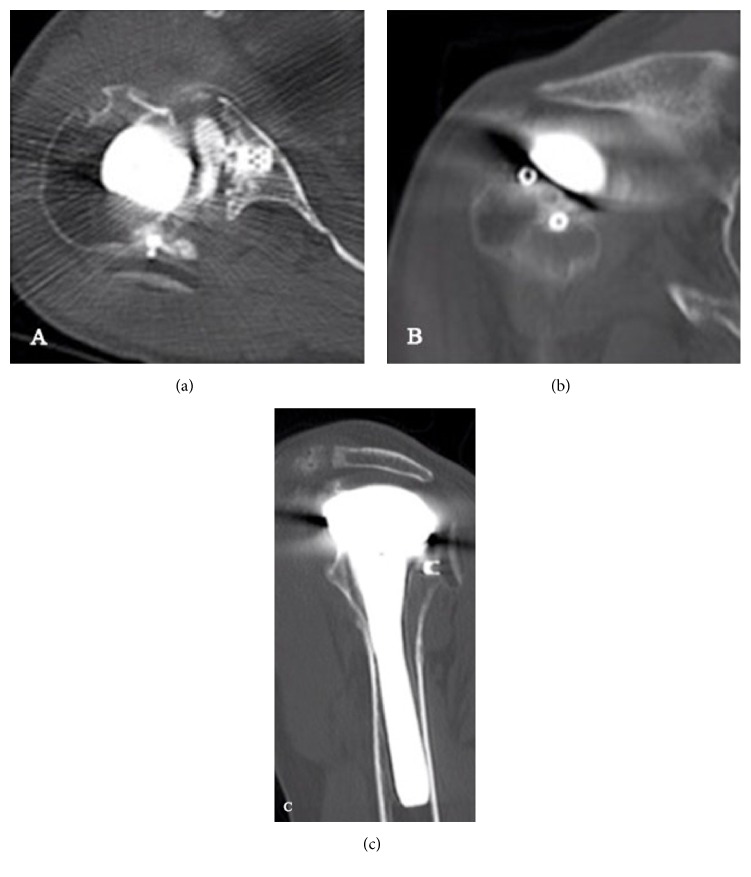
Selected axial (a), coronal (b), and sagittal (c) cuts of CT demonstrating dissociated glenoid component with cage remaining seated in the glenoid.

**Figure 2:**
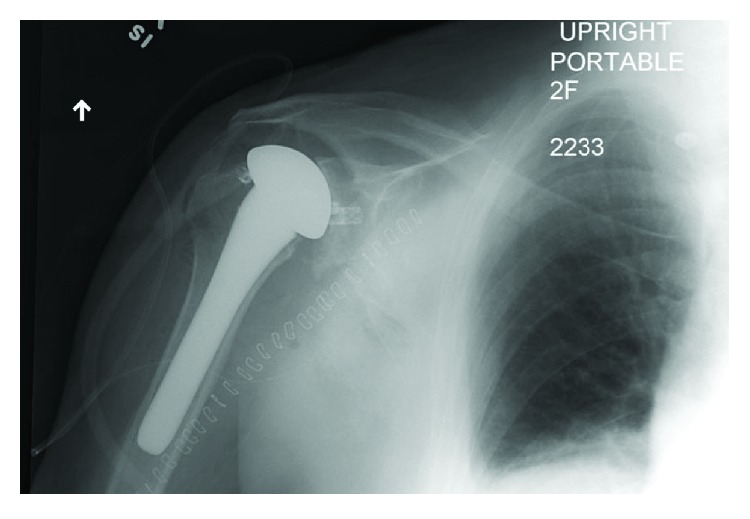
Postoperative X-ray, day of primary TSA.

The patient is a 65-year-old man referred to the senior author's clinic with persistent right shoulder pain and severely restricted range of motion approximately 10 months after a primary anatomic TSA. The indication for the primary procedure was longstanding severe osteoarthritis with an intact cuff. The Exactech Equinoxe (Exactech Inc., Gainesville, FL) shoulder system was used for the index procedure, with a pegged, caged glenoid (model number 314-02-04). Utilizing a standard deltopectoral approach, a 15 mm press fit humeral stem with a 47 × 18 humeral head, offset with a 4.5 mm replicator plate and a large beta curvature, pegged glenoid with cage was implanted. No complications had been noted at the time of the primary procedure. The patient had an uneventful, atraumatic postoperative course, but he failed to progress. His range of motion was severely restricted to about 15 degrees in all planes 8 months after surgery. A CT arthrogram was ordered to evaluate if there was a rotator cuff tear but instead found that the glenoid component had dissociated and was sitting posterior to the humeral head (Figure 1). The polyethylene glenoid component had disengaged from its metallic cage and one of the three metallic peg caps, which remained seated in the glenoid. Close inspection of the postoperative films, as early as those immediately following surgery in the postanesthesia care unit, confirmed that the glenoid had in fact failed acutely (Figure 2).

**Figure 3:**
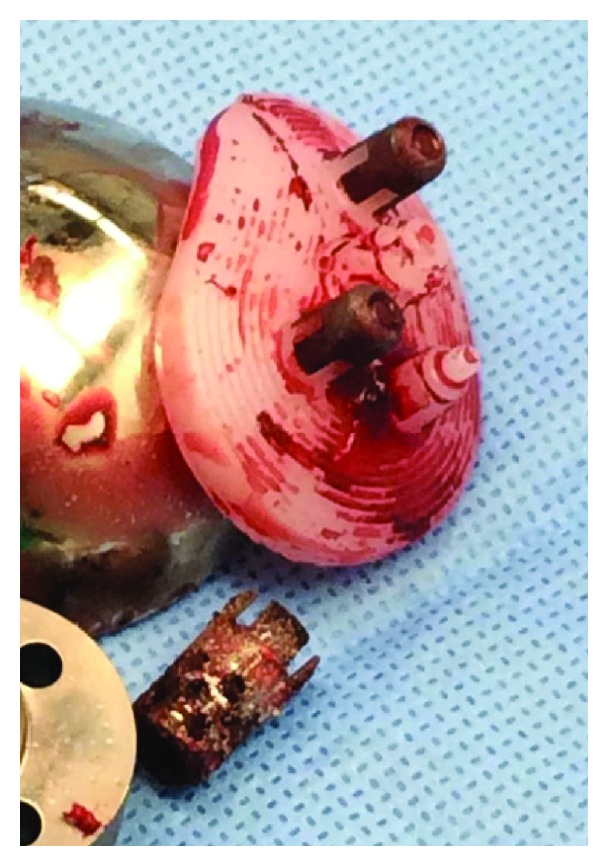
Explanted glenoid component. Note that one of the pegs and the cage have dissociated from the remainder of the glenoid component.

The patient was subsequently referred to the senior author and underwent revision TSA. The glenoid component was retrieved from the posterior capsule. The component failure occurred at the interface between one of the pegs and its metallic cap as well as at the cage component, which had disengaged from the polyethylene (Figure 3). Inspection of the native glenoid revealed a region of proud bone, consistent with inadequate reaming of the glenoid. The retained glenoid cage and peg were extracted and the glenoid was prepared for the new implant. A new pegged glenoid was cemented into place and the humeral head was downsized.

Six months later, the patient was progressing well. He regained 90 degrees of pain-free forward flexion (110 passive) with good rotation. He planned to continue to work on strength and range of motion with physical therapy. However, after the 6 month visit the patient was lost to follow-up. A good faith effort was made to contact the patient, but he could not be reached.

## 3. Discussion

Glenoid loosening is the most common cause of failure of contemporary TSA, comprising 32% of all complications and 7% of all indications for revision [3]. Failure most often occurs years after the initial surgery.

A variety of glenoid designs exist which have had varying degrees of success. A cemented, pegged all-polyethylene glenoid is presently the gold standard [13–17], though it is at risk for aseptic loosening. Because of concern for long-term survival of these implants, metal-backed components—including those utilizing trabecular metal to promote bone ingrowth—have been developed with the hope of improving glenoid fixation. Yet despite a greater percentage of peri-implant radiolucency in cemented all-polyethylene versus metal-backed components (42.5% versus 34.9%), the revision rate remains three times as great for metal-backed components [18]. Metal-backed glenoids have been unsuccessful for several reasons, including polyethylene dissociation from the metal baseplate, increased wear rates resulting in osteolysis of relatively thin polyethylene components, and potential metallosis due to extensive polyethylene wear and secondary metal on metal articulation. Metal-backed glenoids also rely on screw fixation for the metal base plate, which may fatigue and fail over time. Additionally, metal-backed components may cause more stress shielding and associated glenoid bone loss due to significant differences in stiffness between the metal and bone (Young's modulus of <10 GPa in bone versus >100 GPa in metal) [9, 19]. Material properties of all-polyethylene components more closely resemble those of the native glenoid bone, which improves stress transmission and thereby causes less shielding.

Nonetheless, the differences in stiffness between bone and polyethylene (Young's modulus <1 GPa) can create a stress riser across the cement mantle leading to aseptic loosening of all-polyethylene glenoid components. This is the most common point of late failure with this type of implant. To address this issue, glenoid components have been modified to hybrid designs with a central cage component in which bone graft is placed to promote bone ingrowth. The cemented pegs impart rotational stability and initial fixation while the central cage promotes bone ingrowth to improve late fixation. These implants have shown some promise with mid-term results [20–23]. The glenoid component that is the subject of this case report is a variation of this design. What is unique about this particular component is that the central cage is composed of plasma coated titanium to further promote ingrowth. The peripheral pegs are titanium and grit blasted to accommodate potential cementless technique. Based on early results of an Exactech consultant, this glenoid design is showing some promise. At two-year follow-up of 127 TSAs by one surgeon using this system, 3 patients showed radiographic evidence of loosening, 2 of which required revision [23].

It is rare for a component to suffer an acute mechanical failure. Most of the acute glenoid failures described in the literature have been due to late traumatic events. Polyethylene inserts have dissociated from their metal base plates [7–9]. One reported case involved a cemented polyethylene glenoid failure through its pegs with subsequent dissociation into the posterior subcutaneous tissue following a “minor trauma” [6]. In one of the revised failures of the Exactech Equinoxe system noted previously, the central cage locking mechanism failed [23]. There is an additional failure of this system registered with the MAUDE Database (Manufacturer and User Facility Device Experience Database) in which the glenoid component became “prematurely loose” one year after implantation [24].

The cause of acute glenoid failure in the subject of this case report was likely multifactorial and in all likelihood related to inadequate glenoid exposure. The glenoid component failed through the cement-implant interface as well as within the implant itself. The metal cage and one of the three metal peg caps dissociated from the polyethylene and remained seated in the patient's glenoid, while the remainder of the component dissociated posteriorly. The cement mantle failed acutely, which may have been due to premature cement hardening before the glenoid was impacted or improper component positioning secondary to poor exposure. Less likely, the cement may not have adequately set when compression was released and the shoulder reduced. However, with this implant, it has been reported that holding compression while the cement sets is an unnecessary step.

The dissociation of the metallic peg caps and cage from the polyethylene is not without precedence. In the two revised failures of the Exactech Equinoxe system noted previously, the central cage locking mechanism failed as well. Especially for this implant, glenoid exposure is paramount. The component requires “straight line” glenoid impaction. It must be directly perpendicular to the face of the glenoid to prevent damage to the locking mechanism of the central peg as it engages the drilled hole. Failure to do so may disengage the central peg from the polyethylene [23]. This technical point is likely the cause of the failure in this patient.

## 4. Conclusion

Glenoid component failure remains the most common cause of TSA failure and has been the focus of recent implant design modifications. The design rationale behind this hybrid glenoid component is intriguing, but it remains to be seen if it is a durable solution for glenoid failure. Further investigation with long-term outcome and comparative studies are necessary. Regardless of which TSA system is being used, it is imperative that the surgeon achieve adequate exposure for proper component placement. Poor glenoid exposure was likely the biggest factor in the component failure presented here. Delay in detection of failure in TSA can lead to poor outcomes. Postoperative monitoring of the patient's clinical progress and close radiographic evaluation of implant position, glenoid tilt, and peri-implant osteolysis will aid in catching failures early. Ultimately, good implant design, surgical technique with adequate exposure, and vigilant postoperative surveillance give patients the best chance for a successful long-term outcome with TSA.
